# The Mechanical Performance of Re-Bonded and Healed Adhesive Joints Activable through Induction Heating Systems

**DOI:** 10.3390/ma14216351

**Published:** 2021-10-24

**Authors:** Raffaele Ciardiello

**Affiliations:** 1 Department of Mechanical and Aerospace Engineering, Politecnico di Torino, 10129 Turin, Italy; raffaele.ciardiello@polito.it; 2 Inter-Departmental, Multi-Disciplinary Research Center J-TECH@PoliTO, 10129 Turin, Italy

**Keywords:** reversible adhesive joint, thermoplastic adhesive, healing procedure, single lap joint, functional adhesive

## Abstract

This work aims to study the healing potential properties of a reversible thermoplastic adhesive. The adhesive is activable by using induction heating systems that can induce thermal heat in the particles throughout the electromagnetic field so they can melt the adhesive for bonding or separation procedures. The healing procedure consists of damaging single lap joint (SLJ) specimens with quasi-static and fatigue tests and then using an inductor to generate an electromagnetic field able to heat the adhesive to its melting point in order to heal the damaged SLJ specimens. SLJ tests were performed on damaged and healed specimens to assess, respectively, the residual mechanical properties of the damaged specimens and the mechanical properties after healing. SLJ tests showed that the healing procedure can completely recover the joint stiffness of the damaged adhesive joints, a huge part of the maximum shear strength and the SLJ absorbed energy. This work shows also the possibility of re-bonding completely failed or separated SLJs by using the same procedure. The mechanical properties of SLJs after healing and re-bonding are compared to the SLJ compared on virgin specimens to assess the recovered mechanical properties.

## 1. Introduction

Adhesive joints are widely adopted in many industrial sectors especially where composite materials and plastics are used, such as the automotive, naval, and aerospace sectors [1]. Their adoption is due to the ease of application, a more uniform stress distribution compared to screws, bolts, and nuts, their sealant properties, and the possibility of resisting corrosive environments [1]. Although they present these advantages, they present some drawbacks that can limit their adoption, such as the possibility of easily dismantle the joined components when required. This aspect is very important at the end of life of a vehicle due to strict regulations imposed by the governments, especially in Europe, where the Directive of the End of Life vehicle [2,3] imposes that automotive industries have to list all the existing techniques that allow for component dismantling. In addition to dismantling, the separation of adhesive joints is useful also for repair, overhaul, or avoid waste during the bonding operations [4,5]. Lu et al. [5] and Banea et al. [6] reported the newest patented and proposed in literature technologies able to ease the separation processes of adhesive joints. These new and innovative technologies have been studied because the current traditional techniques (the use of chemical agents, heat, or mechanical cut) lead to damage to substrates or components [5,6,7]. Among the techniques presented in [5,6], some of them can be fully reversible since they use thermoplastic adhesives that can be remelted (as compared to thermoset adhesives) and thus they can be used for repair, overhaul or avoid waste during the bonding operations. Although the use of heat can be a valid alternative to separate adhesively bonded components with thermoplastic adhesives, there are some issues that can limit their use. Lu et al. [5] reported that the use of heat can introduce damage to components when the components and the adhesives have similar melting points. Furthermore, huge components cannot be easily adapted in the oven and they need a long time of exposure to be separated. For these reasons, the need to find alternative techniques is crucial to promote the rapid separation of adhesively bonded components. Many studies [5,6,7,8,9,10,11,12,13,14,15,16,17,18,19,20,21] have been presented in the literature regarding the dismantling of the adhesive joints of thermoplastic components. The use of iron oxide nanoparticles dispersed in a holt-melt adhesive that is activable through induction heating has been extensively studied by Ciardiello et al. [7,8,9,10]. These research activities [7,8,9,10] showed that the separation of adhesively bonded joints is feasible by adopting weight concentrations from 3% wt. to 10% wt. This technology presents separation times from 109 s to 13 s, respectively, by applying frequencies of 310 kHz and power of 5.9 kW. The mechanical properties of the adhesive joints were also studied by the same authors [7,8,9,10]. The studies reported an increase of the maximum shear strength (for the substrate made of a copolymer of polypropylene), larger ductility for the adhesive joints prepared with nanoparticles, the same impact resistance, and the same behaviour of the joints after ageing cycles [7,8,9,10]. The principle behind induction heating is quite known [7,8,9,10]. The particles can increase their temperature when immersed in an electromagnetic field, due to the Neel and Brown relaxation effects and hysteresis losses. These Neel and Brown effects are related to the excitation of the particles and the relative motion of the nanoparticles in the thermoplastic adhesive [9]. The heating rapidity of iron oxide particles is also related to a size effect of the nanoparticles that, below 50 nm, exhibit superparamagnetic behaviour that leads to a more rapid increase of the temperature, as Ghazanfari et al. [11] have reported. If the desired effect is just dismantling the adhesive-bonded joints, an alternative technique has been analysed by Banea et al. [13,14,15]. These studies use an inductor to heat substrates made of metal. In this way, thermally expandable particles (TEP) that are embedded in the adhesive can increase their temperature by conduction and the particles can expand. The increase of the particles’ sizes generates voids within the adhesive film that eases the separation of the adhesive joints. Study [14] reported that TEP particles can separate adhesive joints by using particle weights from 5% to 25%. However, TEP particles embedded in the adhesive induce a reduction of the ultimate displacement and shear strengths between 14% to 45% for the adopted concentrations [15]. Vattathurvalappil et al. [16,17] studied a similar system to separate SLJs bonded with a thermoplastic adhesive, acrylonitrile butadiene styrene (ABS). They studied the mechanical and separation properties of an ABS adhesive modified with Fe_3_O_4_ particles by using different weight concentrations between 4% and 20% [16], and found that a weight concentration of 8% is needed to melt the ABS adhesive. On the other hand, the shear strength increased by 8% compared to the joints prepared with the unmodified adhesive in a 16% weight concentration [17]. Although these latter techniques give interesting results for the dismantling of adhesive joints, the possibility to re-bond or heal (i.e., for repairing) these adhesive joints by using the same adhesive was not evaluated. Furthermore, methodologies for evaluating the mechanical properties of a healed adhesive joint and of the re-bonding process itself are not defined in the literature. 

The healing properties of adhesive joints have been studied in only a few works [21,22,23,24,25,26,27]. Most of the studies on adhesive joint healing properties are applied to composite materials [21,22,23]. To my knowledge, the first work on this subject was published by White et al. [21] who embedded microcapsules and catalysts in a composite matrix, such that the propagation of the crack led to the fracture of the capsules, which released a healing agent that can polymerise when in contact with the catalyst, and thus recover the cracks. The work reports a 75% recovery of toughness with a system that is autonomic. The same method has been used also for adhesives by Jin et al. [26], who reported a recovery of fracture toughness of 56% by letting the specimen cure at room temperature. The main limitations of this technique have been put in evidence by Vattathurvalappil et al. [4], and were due to the size of the microcapsules that had to be large enough to provide a good amount of the healing agent. This leads to the relevant inconvenience that microcapsules act as stress concentrators, affecting the structural integrity of the materials prepared with these capsules. Another healing approach for adhesives was used by Li et al. [22,27]. They incorporated thermoplastic particles in a thermoset adhesive and, afterward, the cracked adhesive joints were compressed in a steel frame under heat. The supplied heat liquefied the thermoplastic particles, which would flow and close the cracks. The authors reported the result of the double cantilever beam tests without particles, with particles, and after repeating one, two, and three consecutive healing cycles. The particles led to a reduction of mechanical properties of 5%. This healing procedure was assessed by damaging the join and repeating the healing procedure three different times. A decrement of the maximum peak load of 10%, 15%, and 18% was found after one, two, and three healing cycles respectively. 

A different healing approach was used by Aubert [25]. With this technique, an adhesive is prepared through the reaction of aliphatic diamines and a diepoxy compound containing two Diels–Alder adducts. By means of a warm-up the chemical links disconnect and so the adhesive becomes weak enough to be removed and the elastic modulus is reduced by a factor of 103. Upon cooling, the links reconnect, and the adhesive restores its mechanical properties. The author reported only the results of the performed dynamic mechanical analysis (DMA). The same approach was used by Chen et al. [23] and Bekas et al. [24], who conducted their tests on quasi-static SLJ. The healing leads to a 75% recovery of the failure load measured on virgin joints.

Although some works [4,25,26,27] have been presented in the literature on the healing properties of adhesive joints, there is no commonly adopted methodology to define the healed properties of the adhesive joint, and the small number of research activities present many drawbacks, as discussed above. For this reason, in this work, the mechanical properties of damaged and healed SLJ specimens are analysed together and a rigorous methodology is proposed for the determination of the healed properties of the adhesive joints, together with a new method to heal adhesive joints by electromagnetic fields. The works presented in the literature [25,26,27], to the best of the author’s knowledge, report, in some cases, tests on particular adhesive joints, such as a double cantilever beam (DCB) that are not widely adopted to evaluate the mechanical properties of adhesive joints because usually adhesives are used to withstand shear loads. Furthermore, other works do not report the final value of the adhesive thickness of the tested SLJ after healing, which is an important kind of information since SLJ strength is highly dependent on adhesive thickness, as shown in [9]. For this reason, in this study, specific attention is paid toward the final thickness of the adhesive joints.

In this work, the mechanical properties of adhesive joints made of polypropylene substrates and a polyolefin hot-melt adhesive modified with iron oxide particles were assessed through SLJ tests. Afterward, SLJs were damaged by using either quasi-static SLJ or fatigue tests. Then, damaged specimens were healed using a healing procedure that consisted of heating the damaged SLJ using electromagnetic fields. The electromagnetic fields activated nanoparticles embedded in the SLJ to melt the adhesive and then remake the joints without the need for the external addition of adhesive. When the melting point was reached, a mass of 3.5 kg was placed on the specimen placed in a mould to ensure the pressure needed to restore the joints during the cooling phase. SLJ tests were carried out to assess the mechanical properties of the healed specimens. Besides the healing properties, the re-bond after induction heating, separation and post-SLJ tests were assessed using the same procedure. 

## 2. Materials and Methods

### 2.1. Nomenclature

In the following sections, ND refers to non-damaged SLJ specimens that were adopted as the baseline to compare the mechanical properties of the healed SLJ specimens, D refers to the damaged specimen, SH refers to the healed specimen, FAT refers to the SLJ subjected to fatigue loads (800 cycles in load control in the range 660–220 N), QS refers to an SLJ subjected to quasi-static tests interrupted in the plastic region at 80% of the maximum load, SEP refers to SLJ that were separated by using induction heating system, and CS refers to SLJ that were completely separated with SLJ test. More details about the damaged and separated SLJs are reported in Section 2.2.

### 2.2. Materials and Methods

The adhesive joints used for the experimental tests were obtained by bonding substrates made of a polypropylene copolymer with 10% by weight of talc, (Hifax CB 1160 G1, by Lyondell-Basell Industries, Houston, TX, USA). Rectangular adherends, 100 mm long with cross-sections of 20 × 3 mm, were used as substrates for the experimental tests. The substrates were bonded with Prodas, a polyolefin-based hot-melt adhesive by Beardow Adams (Milton Keynes, United Kingdom), which is a copolymer of polypropylene and polyethylene. The properties of the adhesive are reported in the Table 1 and have been studied in preliminary work [9,10,12]:

The chemical and thermal characteristics of the adhesive used in this work have been studied by Koricho et al. [12]. A preliminary study showed that the adhesive used in this work presents a maximum tensile strength of 1 (±0.1) MPa, an elastic modulus of 4 (±0.1) MPa and a maximum deformation higher than 600% (±40). These results are confirmed by the study [12]. The adhesive was prepared with the addition of 10% weight percent of iron oxide (IO) particles. The mechanical properties of the adhesive joints [9], their resistance to ageing cycles [8], and their separation using induction and microwave systems [10] were studied in preliminary works [10]. 

The IO nanoparticles (Fe_3_O_4_, by Sigma-Aldrich, St. Louis, MO, USA) presented a density of ~5.5 g/cm^3^ and spherical shapes, with an average size smaller than 50 nm. Ciardiello et al. [10] reported that these particles “as received” present agglomerates with an average size of 1 µm. However, extruding the particles with a twin-screw extruder together with the adhesive can reduce the presence of agglomerates and lead to a more uniform particle distribution. 

Thus, the modified adhesive was prepared by using a twin-screw extruder, Haake MiniLab Extruder (Thermo Scientific, Waltham, MA, USA). The pristine adhesive was mixed with IO nanoparticles by melting the adhesive at 190 °C and then adding the particles by mixing the compound with a glass rod. Afterwards, this compound was mixed with the extruder at 100 °C for 10 min at a speed of 120 rpm. This is the time required to obtain a constant value of the mixing torque. The procedure described here has been used by Ciardiello et al. [10] to produce the same masterbatch that was used in the present work. This procedure led to a uniform particle distribution in the adhesive matrix. A scanning electron microscope analysis (SEM) (Zeiss, Jena, Germany) of the same masterbatch was performed by Ciardiello et al. [8,9,10] in preliminary works. The work [10] showed that the extruder used to prepare the masterbatch can uniformly disperse the IO particles in the adhesive and that this method can reduce the presence of the large agglomerates (~1 µm) that were found with the hand mixing method that was used to preliminarily mix the particles before the extrusion process [9,10]. There was no difference in the SEM images before and after induction heating.

Both the mechanical and separation tests were carried out on SLJ specimens, according to the standard procedure for SLJ [9]. The joint preparation was performed using a hot-melt gun (spreading temperature 180°) (Namitech, Pineta di Laives, Italy) and an assembly device to control the adhesive layer thickness and the overlap length of the joint, as done in [9,10]. The adhesive was spread on the lower substrate, then the upper substrate was coupled with the lower using a designed device that used a calibrated screw and some pins to obtain the desired adhesive thickness and overlap, respectively. The pressure along the overlap area was ensured by mass of 3.5 kg applied on the upper substrate. Based on previous experience with these substrates and adhesives [7,8,9,10], the SLJs were prepared using an overlap length of 25 mm and a width of 20 mm. The thickness of the adhesive joints was 1 mm, as suggested by the supplier [10]. All the substrates were cleaned with isopropyl alcohol before joint preparation. The tests were carried out at least 48 h after their preparation.

The SLJ tests were conducted at a constant displacement rate of 5 mm/min using an Instron 8801 hydraulic machine (Instron, Norwood, MA, USA). At least five bonded joints were tested for statistical purposes. The undamaged SLJ presented an adhesive thickness of 1 ± 0.03 mm while the healed and rebonded specimens presented a final adhesive thickness of 0.9 ± 0.03 mm.

The separation tests and healing procedures were carried out with the inductor Heasyheat by Ambrell (Rochester, NY, USA), which presents a maximum power of 10 kW and a frequency range from 10 to 400 kHz. The configuration adopted for the separation tests of the SLJs was the same as those adopted by Banea et al. [13], Ciardiello et al. [7,8,9,10], and Vattathurvalappil et al. [16,17]. A mass of 0.5 kg was used to induce the initial shear sliding when the adhesive melted. However, the separation tests were carried out to obtain the separation of the joint and then perform re-bonding procedure to assess the mechanical properties after joint healing. The separation was carried out on virgin specimen by using a solenoidal coil, as done in [10], electrically excited with a frequency of 315 kHz and a power of 7.0 kW. The virgin joints were separated in a mean time of 13.5 s (±1.2 s). This information is reported here as reference data for comparison purposes.

**Figure 1:**
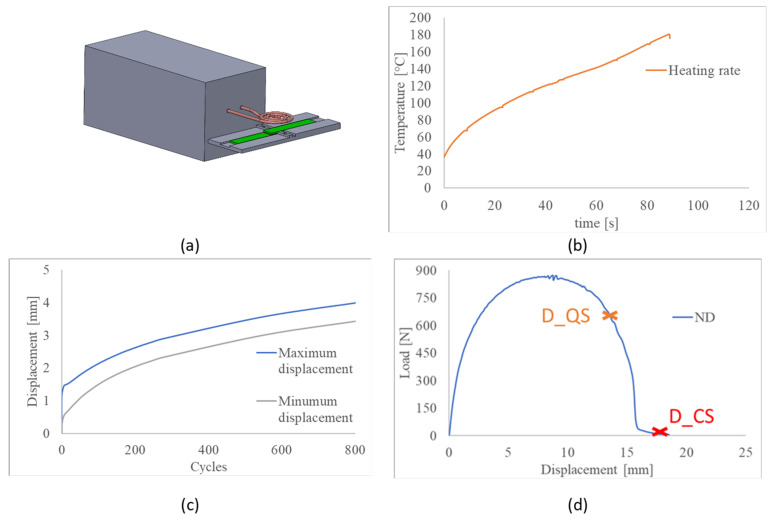
(**a**) Healing configuration; (**b**) heating rate of the SLJ; (**c**) maximum displacement obtained during the fatigue tests; (**d**) points where the test were interrupted for the tests D_QS and D_CS.

For this study, an inductor with a different geometry, a pancake coil, was adopted, using a power of 9.0 kW and a frequency of 240 kHz to heat the adhesive to its melting point in order to re-bond or heal the damaged specimens with the specimens that were separated mechanically or by induction heating, as shown in Figure 1a. The healing procedure was performed as shown in Figure 1a, where the SLJ was heated with the heating rate presented in Figure 1b. Both solenoidal and pancake coils use circulating water to cool down the temperature of the coil and maintain it in the range of 28–35 °C. The solenoidal coil is a three-turn coil with an external diameter of 25 mm; the three turns are equally spaced over a length of 25 mm so that the adhesive overlap of the joint can fit entirely within this length, as shown by [9]. The separation test was performed by placing the SLJ in the centre of the coil. The pancake coil is also made with three turns, and presents an external diameter of 44 mm with turns that are equally spaced. The healing procedure was performed by using an SLJ support to heat the adhesive of the single lap joint along its overlap. The pancake coil was placed 5 mm from the SLJ top surface and the adhesive was heated up to 180 °C, the temperature at which the adhesive is completely melted, and the temperature used to make the undamaged joints. The temperature was measured on the edge of the SLJ, as Ciardiello et al. [9] showed that modified adhesives heated using this system experience uniform heating within the adhesive layer. The distance between the SLJ and the coil was chosen to induce a soft heating rate [9], as shown in Figure 1b. As soon as the adhesive temperature reached 180 °C, two pins in the structure were used to bring the damaged specimen to its original overlap length, 25 mm, and then a mass of 3.5 kg was added to the SLJ to squeeze out the adhesive excess and to make the bond, as performed for the virgin specimens. The damaged specimens presented different overlap lengths due to the sliding effect of the mechanical tests. The temperature of the adhesive was measured by an infrared camera (EIgroup, Bovisio Masciago, Italy), IRtech radiamatic Timage (thermal sensitivity of 80 mK, image acquisition rate of 80 Hz). Damage was induced in two different ways: fatigue tests, and quasi-static tests. Figure 1c shows the damage evolution in the oligocyclic (800 cycles) zone by setting the maximum applied load at 80% of the ultimate load obtained in the quasi-static test (660 N) and the minimum at 220 N. The frequency of the fatigue tests was 2 Hz for all the tests subjected to fatigue loads. The idea was to damage the SLJ specimen by using the same type of load used for the quasi-static test. Finally, the damage induced by quasi-static tests was obtained by performing a SLJ at 5 mm/min and by stopping the test after having reached the plastic region, i.e. after overcoming the maximum peak, at 660 N. Figure 1d shows the point where the test was stopped to induce QS damage (orange point indicated with X) and the complete separation with the SLJ tests (red point indicated with X). In a preliminary activity, transverse impact tests on SLJs in the range of 0.5–2 J were performed to damage the SLJ as performed by Vattathurvalappil et al. [4]. However, the polypropylene substrates were too flexible to cause damage within the adhesive with these tests. The result was a plastic deformation of the substrates and, for this reason, SLJs were instead damaged with quasi-static and fatigue tests.

## 3. Results

### Mechanical Test Results

**Figure 2:**
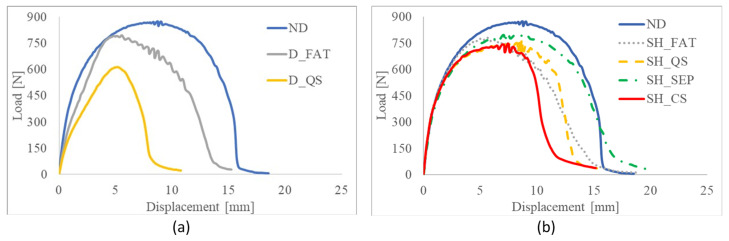
(**a**) Comparison among the undamaged and damaged SLJ tests; (**b**) comparison among undamaged, healed, and rebonded SLJs.

Figure 2 presents the mechanical behaviours of adhesive joints without damage, damaged, healed, and re-bonded. The blue curve is related to the not-damaged (ND) specimens and presents the maximum load. On the other hand, the damaged SLJs with fatigue load (D_FAT) and quasi-static load (D_QS), presented reduced mechanical properties. In particular, Figure 2a shows that the specimens damaged with quasi-static load presented a significant reduction of the maximum load, maximum displacement, and adhesive joint stiffness, the latter being represented by the initial slope of these curves. The fatigue test led to a reduction of the mechanical properties, but in this case it was less significant than the SLJs damaged with quasi-static load. However, the maximum load decreased and moved leftwards, the stiffness was reduced, and a lower ductile behaviour compared to the baseline was exhibited. 

Figure 2b shows the healed and re-bonded curves together with the baseline (ND) for a direct comparison of the effects of the healing process. It is possible to note that all the joints fully recovered their initial stiffness. Figure 2b shows that the adhesive joints that recovered the most of its baseline maximum load were the SLJs separated with induction heating and then rebonded. Although the maximum load of the joints separated with induction heating was lower compared to the baseline joint, the ductile behaviour was recovered. The reason could have been due to the separation surfaces of the joints both being cohesive, as shown at the end of this section. Furthermore, the joints separated with quasi-static tests and the joints completely separated by SLJ tests exhibited similar trends in the initial part and up to the maximum load. Afterward, the displacement of completely separated joints was about 3 mm lower compared to the ones separated with quasi-static tests. However, a direct comparison between the quasi-static damaged SLJs in Figure 2a and the joints damaged in the same way and then healed shows that the stiffness was totally recovered. Further, the maximum load, after a decrement of about 280 N, recovered about 150 N, and the final displacement, in the proximity of the maximum drop, was almost doubled after healing. Finally, the joints healed after fatigue tests recovered only their original stiffness, while the maximum load and the right tail relative to the ductile behaviour of the joints, did not exhibit significant recovery. This could be related to the separation surface, which, as a consequence of the dynamic fatigue load, led to an extension of the adhesive failure zone.

**Figure 3:**
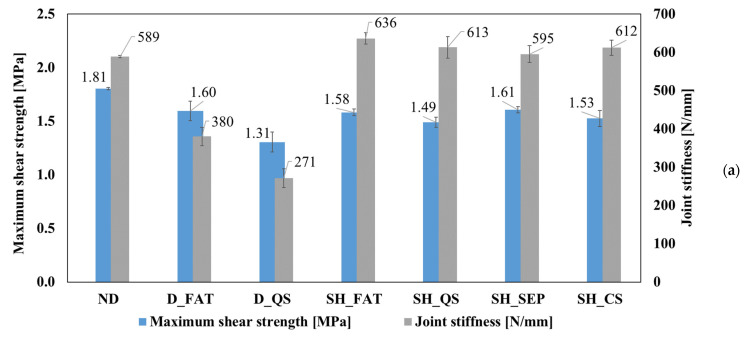

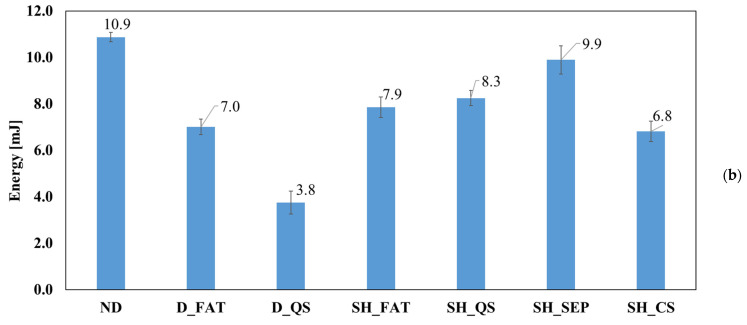
(**a**) Maximum shear strengths and joint stiffnesses of the undamaged, damaged, healed, and rebonded SLJ tests; (**b**) maximum absorbed energy of the undamaged, damaged, healed, and rebonded SLJ tests.

Figure 3a collects in one diagram all the relevant results obtained from the performed test campaign and thus permits us to make a clearer comparison of the results. In particular, it reports the values of the maximum shear strengths and the joint stiffnesses of the damaged, healed, and rebonded adhesive joints. The bar chart shows that the average values of the stiffnesses, calculated in the first linear trend of the force-displacement curves, of all the healed and rebonded joints is higher than those of the adhesive joint of the baseline, and thus the stiffness was totally recovered after the adopted healing process. The increment of the stiffness could be explained as a consequence of the slightly lower adhesive thickness reached after the re-bond and healing procedure, which was 0.9 mm instead of the original 1 mm. Furthermore, the bar chart in Figure 3a shows that the adhesive joints damaged with quasi-static load, after healing, presented a value of stiffness that was 130% higher compared to the damaged joint one, while the increment of the stiffness was 70% in the case of the healed joints damaged by fatigue loads. On the other hand, the maximum average shear strengths of the damaged, healed and rebonded joints were always lower compared to the non-damaged joints. 

Figure 3b shows the average values of the deformation energy computed by using the force-displacement curves of SLJ tests. This is representative of the absorbed energy during the test. Of course, the absorbed energy for the baseline specimens was the highest, since it presents the maximum load and displacement. On the other hand, the absorbed energy values of SLJ exhibited by the damaged specimens show that the reduction was 35% for SLJs subjected to fatigue loads and 65% for the SLJs damaged with quasi-static loads. Further, after healing, the absorbed energy increased by 12% in case of fatigue load while it increased by 90% in the case of quasi-static damage, SH_QS. The SLJ specimen separated with induction heating recovered almost (−9%) all the total energy of the baseline specimens. On the other hand, the specimens healed after complete separation recovered 62% of the original energy. Vattathurvalappil et al. [4] showed that a similar healing procedure conducted on transverse low-impact tests led to a reduction of 33% of the maximum shear strength. After healing, the maximum shear strengths were recovered to 90% of the baseline. However, the final adhesive thickness, in this case, was 0.5 mm (initial thickness 1.0 mm).

**Figure 4:**
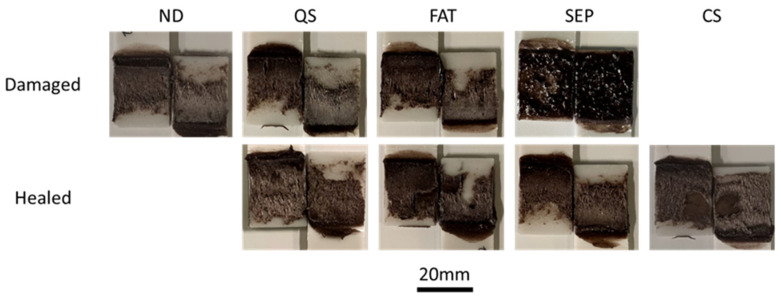
Failure surfaces of the substrates after SLJ tests for the undamaged, damaged, healed, and rebonded specimens.

Figure 4 shows the failure surfaces after SLJ tests for the undamaged and damaged specimens in the first row. Further, the failure surface after the separation by induction heating is reported in the first row of Figure 4. The second row of Figure 4 reports the failure surfaces after the SLJ tests conducted on the healed and rebonded SLJ specimens. The baseline specimen failure surface was very similar to the failure surfaces obtained by Ciardiello et al. [7,8,9,10] in previously published works. The failure surface is always mixed, both adhesive and cohesive, although the adhesive failure type is limited to the edge, as can be seen for the failed ND specimen. Mixed failure surfaces for hot-melt thermoplastic adhesives have also been reported by different authors [4,12,16,28,29] and also by preliminary works carried out by the author [7,8,9,10,30,31]. These works [7,8,9,10,30,31] showed that adhesive failure can be reduced, but not avoided, with substrates that are used in the industrial sector. Specimens damaged with quasi-static loads (QS) displayed no significant differences with respect to the baseline specimens, which was expected since the quasi-static test was interrupted at 80% in the plastic zone and then resumed to complete separation. As shown in Figure 1d, the SLJs were interrupted at 660N and then SLJ tests were conducted on damaged specimens. After healing, the specimens damaged with quasi-static loads presented a larger cohesive area, especially for the specimen that is on the left. This led to higher values of maximum strength and the ultimate displacement. On the other hand, the fatigue test led to a slightly different fracture surface. The failure surfaces of the specimens damaged with fatigue (FAT) tests present a larger portion of adhesive failure close to the edges compared to the baseline specimen, in which a thin layer of adhesive was also present close to the edge. Further, the FAT specimens also presented a small portion of adhesive failure in the central part of the specimen. This part was also present on the healed specimens after the fatigue tests, meaning that the healing procedure was not able to strongly bond this area. This behaviour could be due to the dynamic nature of the load, since Ciardiello et al. [30] showed that SLJs subjected to dynamic loads in the range 1.5–3.7 m/s, led to adhesive failure. However, the healed specimen after the fatigue test presented a larger portion of cohesive area on the left specimen, which led to higher values of maximum strength compared to FAT. The specimen completely separated with induction heating (SEP) shows a separation surface that looked homogenous and was the same as reported by Ciardiello et al. [7]. The healed SEP specimen presented a failure surface that was more cohesive with respect to the specimens healed after quasi-static and fatigue loads, and this led to a higher maximum load. This led to the highest value of shear strength among the healed specimens, and this could have been due to the contact between the two damaged surfaces before healing being more homogeneous, compared to the other configuration. Finally, the failure surfaces of the specimens completely separated with the SLJ test and then healed are presented. These observations were obtained by rebonding the baseline specimen after the test. The failure surfaces are similar to the healed specimens after damage with quasi-static loads (QS) and they presented a similar value of shear strength.

## 4. Conclusions

One of the relevant points in the development of adhesive joints is related not only to the dismounting possibility, but also to rebonding after repair. The present study is based on the already-developed technology of dismounting through the use of properly conceived electromagnetic fields when sensitive adhesives are adopted. The healing and rebonding properties of damaged and completely separated adhesive joints were analysed in this work. 

The performed study dealt with polypropylene substrates bonded with hot-melt adhesive, both of which are used in the automotive industry. Standard SLJs used to perform the mechanical tests, and it is expected that the obtained results can be extended to real-size parts. Induction heating activates iron oxide nanoparticles embedded in the adhesive matrix and can melt the adhesive, allowing for the healing and rebonding of SLJ specimens. 

The mechanical tests showed that, although the joint siffnesses of the joined specimens were reduced consistently after the induced damage due to the fatigue and quasi-static tests, by 35 and 54%, respectively, the joint stiffnesses after healing were completely recovered. The damage induced with fatigue or quasi-static loads led to a relevant reduction of the shear strength of the adhesive joints. The adopted healing procedure was able to recover a huge part (of the order of magnitude of 90%) of the original shear strength. The damage induced with fatigue or quasi-static loads led to a reduction of energy absorption capability at the failure points of 40 and 65%, respectively. The healing procedure adopted in this work was able to recover a huge part of SLJs’ absorbed energy, with a final value of energy absorption at failure being from a minimum of 15% to a maximum of 120% higher when compared to the damaged specimens. 

Furthermore, re-bonding after separation by means of induction heating and quasi-static tests was possible. Although the maximum shear strength of the rebonded specimens was lower with respect to the new one, there was a recovery of at least 85% for the healed and rebonded SLJ. This makes the adhesive joint technology usable after damage or separation.

## Figures and Tables

**Table 1:** Properties of the polyolefin adhesive [9,10,12]. *

Density	0.98 g/cm^3^
Open time	30 s
Softening point	145 °C
Glass transition temperature	−16 °C
Initial thermal degradation temperature	210 °C
Viscosity at 180 °C (S. 27/5 rpm) (BA QA102)	22–28 Pa·s
Melting temperature (Initial–Final)	124 °C–155 °C

* [9] Reproduced with permission from R. Ciardiello, *International Journal of Adhesion and Adhesives*; published by Elsevier, 2019. [10] Reproduced with permission from R. Ciardiello, *Composite Structures*; published by Elsevier, 2020. [11] Reproduced with permission from G. Belingardi, *International Journal of Adhesion and Adhesives*; published by Elsevier, 2016.

## Data Availability

The data presented in this study are available on request from the corresponding author.
